# Feasibility of CMR Imaging during Biventricular Pacing: Comparison with Invasive Measurement as a Pathway towards a Novel Optimization Strategy

**DOI:** 10.3390/jcm12123998

**Published:** 2023-06-12

**Authors:** Luuk H. G. A. Hopman, Alwin Zweerink, Anne-Lotte C. J. van der Lingen, Marthe J. Huntelaar, Mark J. Mulder, Lourens F. H. J. Robbers, Albert C. van Rossum, Vokko P. van Halm, Marco J. W. Götte, Cornelis P. Allaart

**Affiliations:** Department of Cardiology, Amsterdam UMC, De Boelelaan 1118, 1081 HV Amsterdam, The Netherlands; l.hopman@amsterdamumc.nl (L.H.G.A.H.);

**Keywords:** cardiac resynchronization therapy (CRT), cardiovascular magnetic resonance (CMR), dyssynchrony, biventricular pacing

## Abstract

Objectives: This prospective pilot study assessed the feasibility of cardiovascular magnetic resonance (CMR) imaging during biventricular (BIV) pacing in patients with a CMR conditional cardiac resynchronization therapy defibrillator (CRT-D) and compared the results with invasive volume measurements. Methods: Ten CRT-D patients underwent CMR imaging prior to device implantation (baseline) and six weeks after device implantation, including CRT-on and CRT-off modes. Left ventricular (LV) function, volumes, and strain measurements of LV dyssynchrony and dyscoordination were assessed. Invasive pressure–volume measurements were performed, matching the CRT settings used during CMR. Results: Post-implantation imaging enabled reliable cine assessment, but showed artefacts on late gadolinium enhancement images. After six weeks of CRT, significant reverse remodeling was observed, with a 22.7 ± 11% reduction in LV end-systolic volume during intrinsic rhythm (CRT-off). During CRT-on, the LV ejection fraction significantly improved from 27.4 ± 5.9% to 32.2 ± 8.7% (*p* < 0.01), and the strain assessment showed the abolition of the left bundle branch block contraction pattern. Invasively measured and CMR-assessed LV hemodynamics during BIV pacing were significantly associated. Conclusions: Post-CRT implantation CMR assessing acute LV pump function is feasible and provides important insights into the effects of BIV pacing on cardiac function and contraction patterns. LV assessment during CMR may constitute a future CRT optimization strategy.

## 1. Introduction

Cardiac resynchronization therapy (CRT) is a well-established therapy for patients with drug-refractory heart failure (HF) presenting with systolic left ventricular (LV) dysfunction and a broad QRS complex, typically a left bundle branch block (LBBB) [1]. To date, CRT is hampered by a relatively high rate of non-response. Approximately 30–50% of CRT patients show no improvement in pump function or clinical symptoms after device implantation [2,3]. Device optimization is a promising strategy to improve therapeutic benefits in these patients, although a non-invasive and reproducible clinical tool is currently lacking [4].

Cardiac magnetic resonance (CMR) imaging has emerged as a highly accurate and reproducible imaging technique which can offer valuable information for CRT screening, including the assessment of the left ventricular (LV) ejection fraction, as well as scar quantification and localization using late gadolinium enhancement (LGE) [5]. On the other hand, the use of CMR for post-implant CRT evaluation has been limited due to safety concerns and potential image quality issues.

Recently, CMR conditional CRT devices with approval for use in the magnetic resonance imaging (MRI) environment have been introduced [6]. The development of these CMR conditional CRT devices now allows for patient follow-up to be performed during a CMR exam. Importantly, this will result in a better understanding of the mechanisms underlying the success of CRT and may facilitate the identification of CRT patients who can benefit from individual device optimization, which may also be performed using CMR imaging [7].

The objective of this pilot study is to assess the feasibility of CMR imaging after the implantation of a CRT with defibrillator function (CRT-D), and to detect volumetric and functional changes when BIV stimulation is either turned on or off. Moreover, acute changes in LV function are related to invasively assessed acute pump function changes measured using a conductance catheter, which is considered the gold standard for acute pressure–volume analysis.

## 2. Materials and Methods

This prospective single-center pilot study was conducted according to the principles outlined in the 1964 Declaration of Helsinki and its subsequent amendments. The collection and management of data was approved by the local medical ethics committee (VU University Medical Center, Amsterdam, The Netherlands, protocol number 2016.032). Written informed consent was obtained from all individual participants included in the study.

### 2.1. Study Population

Ten patients referred for CRT-D implantation were enrolled in the study between February 2019 and December 2021. All patients had a class 1 indication for CRT-D therapy, according to the 2013 ESC guidelines: a New York Heart Association (NYHA) chronic heart failure classification of II, III, or ambulant IV, with an intrinsic QRS width ≥ 120 ms with LBBB, and a left ventricular (LV) ejection fraction ≤ 35%, despite optimal medical therapy [8]. Exclusion criteria for study participation included severe renal insufficiency, with a glomerular filtration rate < 30 mL/min/1.73 m^2^, significant rhythm abnormalities (atrial fibrillation, atrial flutter, atrial tachycardia, or frequent atrial/ventricular extra systole), pacemaker dependency, artificial aortic valve, aortic valve stenosis, or the impossibility to undergo a CMR scan with a gadolinium contrast agent.

### 2.2. Study Design

Patients underwent a CMR scan for LV function and fibrotic tissue assessment prior to device implantation. Subsequently, device implantation was performed by experienced operators in accordance with local and international guidelines. Patients were implanted with a CMR conditional CRT-D system and leads (CRT-D: Iperia 7 HF-T, RA lead: Safio S 53 proMRI, RV lead: Protego S65 proMRI, LV lead: Sentus proMRI, Biotronik, Berlin, Germany). After device implantation, a six-week waiting period was maintained and subsequently, the follow-up CMR examination was performed. The follow-up CMR examination consisted of a two-stage scanning protocol: during BIV pacing (CRT pacing on), in MRI mode, and during intrinsic rhythm (CRT pacing off). On the same day, patients underwent invasive pressure–volume (PV) loop assessment using a conductance catheter at intrinsic rhythm and during BIV pacing (matching the CRT settings during CMR). Conductance catheter measurements are considered the gold standard invasive reference method for the assessment of acute hemodynamic response during CRT [9,10].

### 2.3. CMR Acquisition Protocol

All scans were performed with a 1.5 Tesla MRI system (Siemens AVANTO or SOLA, Erlangen, Germany) using a 32-channel array coil. The pre-device implant CMR protocol included balanced steady-state free precession (SSFP) cine imaging in long axis (two-chamber, three-chamber, and four-chamber views) and short axis orientations (5 mm slice thickness, without gaps, covering the entire LV). LGE imaging was performed for the assessment of myocardial scarring/fibrosis.

The post-device implant CMR protocol involved two stages, during which the device was programmed to the MRI-safe mode. First, BIV pacing during the CMR scan was performed with the CRT-D in DOO mode (10 beats per minute above intrinsic rhythm) with the use of a fixed atrioventricular (AV) delay set to 100 ms and simultaneous biventricular pacing. During this first stage, cine long axis and short axis acquisition was performed using a spoiled gradient echo sequence. Thereafter, the patient was moved out of the scanner, and the BIV pacing function was turned off (intrinsic rhythm). The above mentioned imaging sequence was repeated, and finally, LGE imaging was performed after 0.15 mmol/kg contrast administration (Dotarem^®^, Guerbet, Roissy, France) in short axis views copying cine orientations. A detailed CMR protocol is presented in the Supplemental Materials (Supplemental Figure S1).

### 2.4. Image Analysis

#### 2.4.1. Image Quality

Post-device implantation CMR images were evaluated by pre-defined criteria to assess the quality of the short axis cine and LGE images (Supplemental Figure S2). Image artefacts were rated on a 4-point scale (1. no artefacts, 2. limited artefacts, 3. substantial artefacts, 4. extensive artefacts). The image quality was rated by two experienced CMR readers (L.R. and L.H.).

#### 2.4.2. Volume Assessment

Cine image analysis was performed using Circle CVI^42^ (version 5.13, Circle Cardiovascular Imaging, Inc., Calgary, AB, Canada). An assessment of the cardiac dimensions and function was performed on the short axis cine images. Endo- and epicardial contouring was performed using the automatic contouring algorithm, and manual adjustments were made, if necessary. End systolic and end diastolic volumes (ESV, EDV), stroke volume (SV), and LV ejection fraction (LVEF) were evaluated.

#### 2.4.3. Deformation Assessment

A quantitative deformation assessment was performed on cine images using Circle CVI^42^ feature tracking software, excluding the LV outflow tract slice(s). Global LV longitudinal strain (GLS), circumferential strain (GCS), and radial strain (GRS) parameters were quantified. To assess LV dyssynchrony and dyscoordination, circumferential peak_septal_ - peak_lateral_ delay, as an absolute value, and systolic rebound stretch of the septum (SRS_septal_) were calculated on the mid-LV slice [11].

### 2.5. Invasive Hemodynamic Measurements

After CMR imaging, on the same day, invasive hemodynamic measurements were obtained. A conductance catheter (CD Leycom, Zoetermeer, The Netherlands) was placed in a stable position in the LV apex to obtain PV loops. PV loops were recorded during intrinsic rhythm and BIV pacing (identical pacing settings to those used during CMR). Approximately 60 representative cardiac cycles were averaged, disregarding all inappropriate beats (i.e., extra systoles) with the use of Conduct NT software (version 3.18.1, CD Leycom, Zoetermeer, The Netherlands). CMR-derived LV volumes during intrinsic rhythm were used for the conductance catheter calibration of volumes recorded during intrinsic rhythm. Consequently, the effects of BIV pacing on LV function were quantified by EDV, ESV, SV, and EF.

### 2.6. Statistical Analysis

The results are presented as mean ± standard deviation, for normally distributed data, and median with interquartile range (IQR), for data with a non-normal distribution. Between-group comparison was performed using paired Student’s *t*-tests. Pearson’s correlation was used to quantify associations between continuous variables. Agreement between measurements was assessed by intraclass correlation coefficients (ICCs). ICCs for absolute agreement of single measurements were estimated using a two-way random effect model. Data were considered statistically significant if the *p*-value was <0.05. Statistical analysis was performed using SPSS Statistics v26 (IBM Corporation, Armonk, NY, USA).

## 3. Results

A total of 10 patients were enrolled in the study (mean age 70 ± 7 years, 9 male). Three patients showed a non-ischemic LGE pattern on CMR, three patients showed ischemic sub-endocardial/transmural LGE, and four patients exhibited no LGE (Table 1). The baseline CMR scan was performed 17 (IQR: 6–20) days prior to CRT-D implantation, and the follow-up CMR scan was performed 50 (IQR: 44–55) days after the implantation of CRT-D. None of the patients experienced complications during the CMR examination, and the device evaluation before and after the scan showed unchanged device parameters. At baseline, the mean QRS duration was 167 ± 15 ms, and at follow-up during BIV pacing, it was 147 ± 10 ms (*p* = 0.83). This change in QRS duration did not significantly relate to a change in LV ESV, LV EDV, or LVEF between baseline and follow-up during BIV pacing (r = −0.48, *p* = 0.17; r = −0.16, *p* = 0.66; r = 0.34, *p* = 0.34, respectively).

### 3.1. CMR Scan Quality

**Figure 1:**
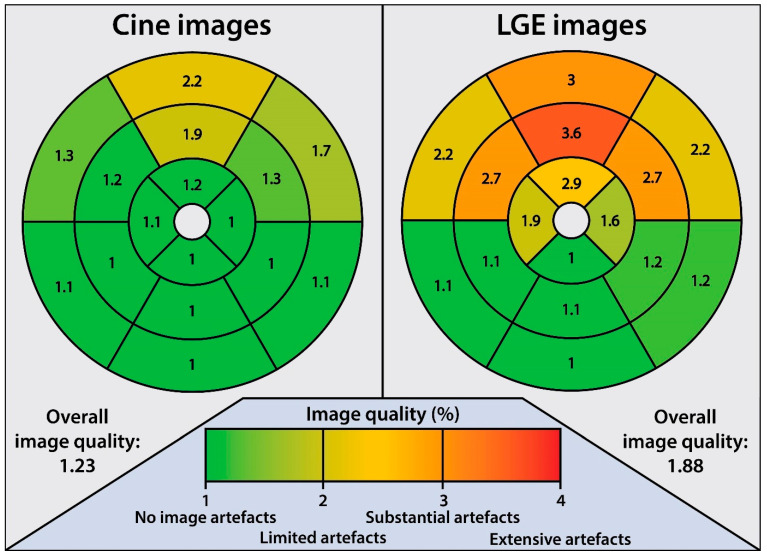
Post-implant CMR image quality. Image quality score in CRT-D patients per segment assessed on short axis images. 1: No image artefact at all, and no limitation in image interpretation. 2: Good image quality, with limited artefacts. 3: Poor image quality, with substantial artefacts. 4: Meaningful image assessment impossible due to extensive artefacts.

Detailed results regarding assessment of artefact extent on the cine and LGE images are given in Figure 1 and Table S1. The CMR scan quality was deemed good for the cine images, although in some patients, the assessment of the cine images was slightly impaired in the LV anterior basal and mid segments (Figure S3). In conventional LGE images, hyperintensity artefacts caused by the CRT-D generator were predominantly observed in the LV anterior, anterolateral, and anteroseptal segments, hampering image interpretation in those areas.

### 3.2. CMR Characteristics at Baseline and Follow-up during CRT-off

**Figure 2:**
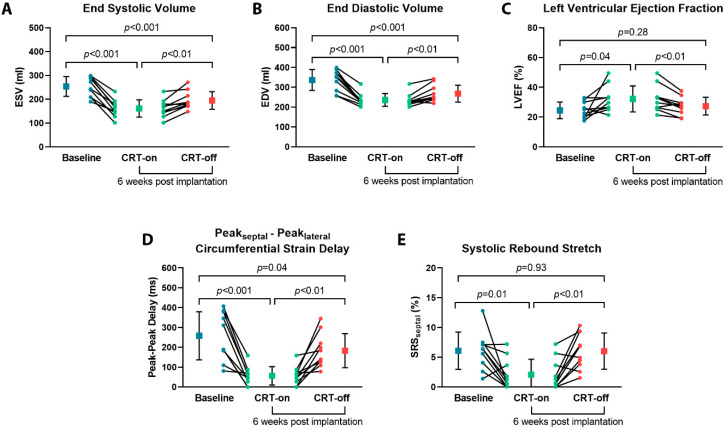
LV volumes and function assessed by CMR at baseline and during follow-up. Comparison of CMR parameters (**A**) end systolic volume, (**B**) end diastolic volume, (**C**) left ventricular ejection fraction, (**D**) peak_septal_ - peak_lateral_ delay, and (**E**) systolic rebound stretch at baseline and follow-up during biventricular pacing and intrinsic rhythm.

At baseline, prior to CRT implantation, the LV dimensions were enlarged (ESV; 253.8 ± 41.3 mL and EDV; 336.9 ± 52.8 mL) and the LV systolic function was impaired (LVEF; 24.6 ± 5.6%). At follow-up during intrinsic rhythm (CRT-off), LV ESV and LV EDV were decreased, as compared to baseline measurements (ESV: 194.9 ± 37.1 mL, *p* < 0.001, and EDV: 268.1 ± 42.3 mL, *p* < 0.001), but no significant improvement in LVEF was observed (27.4 ± 5.9%, *p* = 0.28). Moreover, there was no improvement in septal systolic rebound stretch (6.1 ± 3.1% vs. 6.0 ± 3.0%, *p* = 0.93) (Figure 2 and Table 2).

### 3.3. BIV Pacing Turned on vs. off during Follow-up

**Figure 3:**
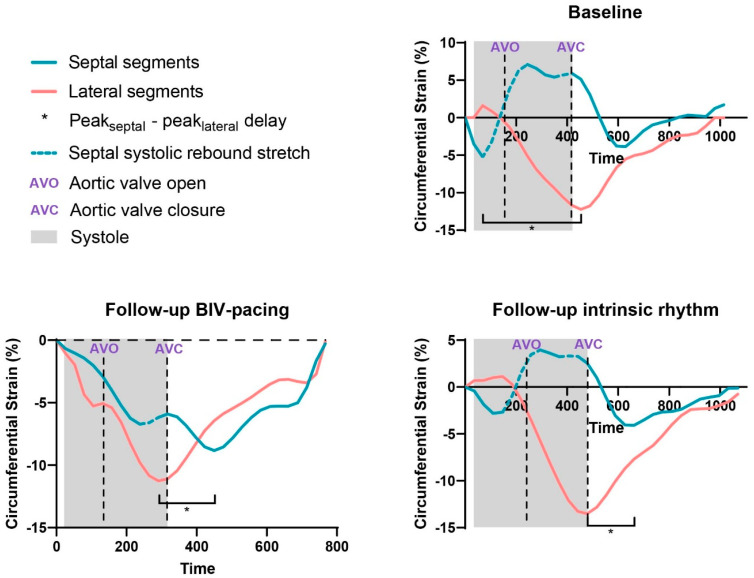
LV circumferential strain assessed using CMR at baseline and follow-up. Left ventricular (LV) circumferential strain curves of the lateral (red) and septal (blue) wall at baseline and follow-up during biventricular (BIV) pacing and intrinsic rhythm. Figures explain the assessment of peak_septal_ - peak_lateral_ circumferential strain delay and systolic rebound stretch. Strain curves of the septal and lateral wall segments display an opposite movement (dotted line) in systole (determined by cine imaging of the LV in the three-chamber view) during follow-up intrinsic rhythm (CRT-off) and at baseline.

Compared to the intrinsic rhythm during the follow-up scan, BIV pacing showed an additional decrease in LV ESV and EDV (ESV: 194.9 ± 37.1 mL vs. 161.4 ± 36.3 mL, *p* < 0.01, and EDV: 268.1 ± 42.3 mL vs. 236.2 ± 31.8 mL, *p* < 0.01) and an increase in LVEF (27.4 ± 5.9% vs. 32.2 ± 8.7%, *p* < 0.01, respectively) (Figure 2 and Table 2). No differences in LV global strain parameters were found, except for a decrease in global longitudinal strain during CRT-on (−7.9 ± 2.2% vs. −6.2 ± 1.6%, *p* = 0.02). Regional dyssynchrony and regional dyscoordination significantly improved at BIV pacing compared to intrinsic rhythm (peak_septal_ - peak_lateral_: 57 ± 46 ms vs. 183 ± 86 ms, *p* < 0.01 and SRS_septal_: 2.1 ± 2.6% vs. 6.0 ± 3.0%, *p* < 0.01). A typical example of regional dyssynchrony and regional dyscoordination assessment is presented in Figure 3.

### 3.4. Invasive Volume Measurements

**Figure 4:**
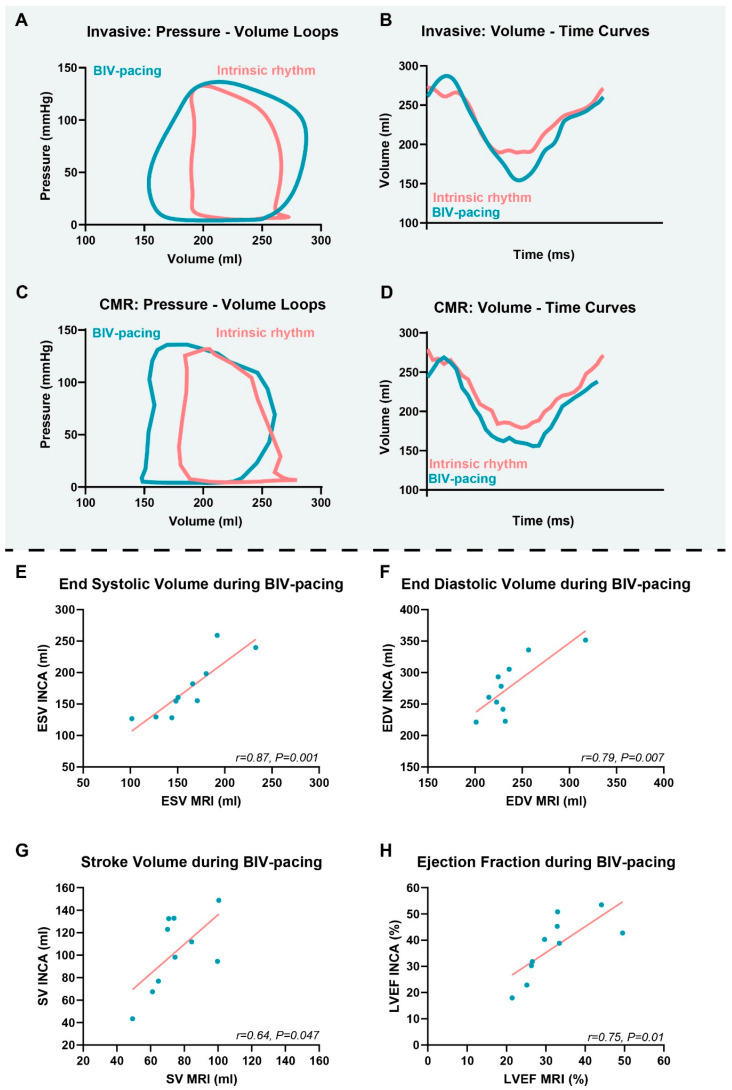
Relationship between invasively and CMR-measured LV volumes and function. In panel (**A**,**B**), a patient example of invasively obtained pressure–volume loops and volume–time curves during BIV pacing and intrinsic rhythm are displayed. Combining the invasively obtained pressures with the MRI volumes, we reconstructed pressure–volume loops and volume–time curves during BIV pacing and intrinsic rhythm (**C**,**D**). The invasive measurements are calibrated using the CMR volumes at intrinsic rhythm. Therefore, the correlations between invasively measured (conductance catheter) and non-invasively measured (CMR) indices (ESV (**E**), EDV (**F**), stroke volume (**G**), and LVEF (**H**)) are shown for BIV pacing.

In Figure 4, the invasively obtained PV loops and volumes are presented (Figure 4A,B). As a proof of concept, an invasively obtained LV pressure curve was matched with an LV volume curve derived from CMR to reconstruct a PV loop (Figure 4C,D). All LV parameters assessed using invasive PV loop measurements during BIV pacing (i.e., ESV, EDV, SV, and EF) were significantly correlated with the LV parameters assessed during BIV pacing in the MRI (ESV: r = 0.87, *p* = 0.001; EDV: r = 0.79, *p* = 0.007; SV: r = 0.64, *p* = 0.047; EF: r = 0.75, *p* = 0.01) (Figure 4E–H). Agreement between the invasively measured LV function and CMR assessed LV function was poor for EDV and SV, and moderate-good for ESV and EF (EDV: ICC = 0.49; SV: ICC = 0.33; ESV: ICC = 0.82; EF: ICC = 0.65).

## 4. Discussion

This pilot study demonstrated that post-implantation CMR imaging can be used to assess the effects on cardiac pump function during BIV pacing in patients implanted with a CRT-D, despite minor image artefacts originating from the generator. The quality of post-implant images was deemed good for cine acquisitions, allowing for reliable assessment of LV volumes and regional wall strains. However, significant device-related artefacts were observed, predominantly in the anterior wall, during LGE imaging, rendering these segments unanalyzable. A decrease in LV ESV and EDV between baseline and follow-up provides evidence of reverse LV remodeling. Moreover, acute hemodynamic improvement was observed upon turning on BIV pacing at follow-up. Furthermore, The ability to detect improvement and deterioration of cardiac function during different CRT settings was validated using invasive hemodynamic measurements. Proving the feasibility of CMR to measure LV pump function during CRT paves the way for using CMR for CRT optimization in the future.

### 4.1. CMR Imaging in CRT-D Patients

CMR imaging is currently considered the gold standard for the non-invasive assessment of ventricular function, mechanics, and detection of scar tissue [12,13]. The impact of CMR assessment on patient selection for CRT and the prediction of CRT response is evident [14,15]. Identifying scar tissue is critical for appropriate LV lead placement over viable myocardium, and measures of dyssynchrony predict clinical outcomes of CRT [5,15,16]. Potentially, CMR can also be used for the evaluation of resynchronization effectiveness and patient monitoring after CRT implantation. However, the reliable evaluation of CRT response has been limited by device-related imaging artefacts and the lack of a BIV pacing option in the MRI-safe mode.

Recent advances have led to the opportunity to safely perform MRI scans in CRT-implanted patients during BIV pacing, and developments in MR sequences have significantly reduced artefacts arising from the generator and leads. These high-quality cine images are essential for the precise quantification of ventricular hemodynamics, including strain, and evaluation of resynchronization effectiveness and patient monitoring after CRT implantation [17].

**Figure 5:**
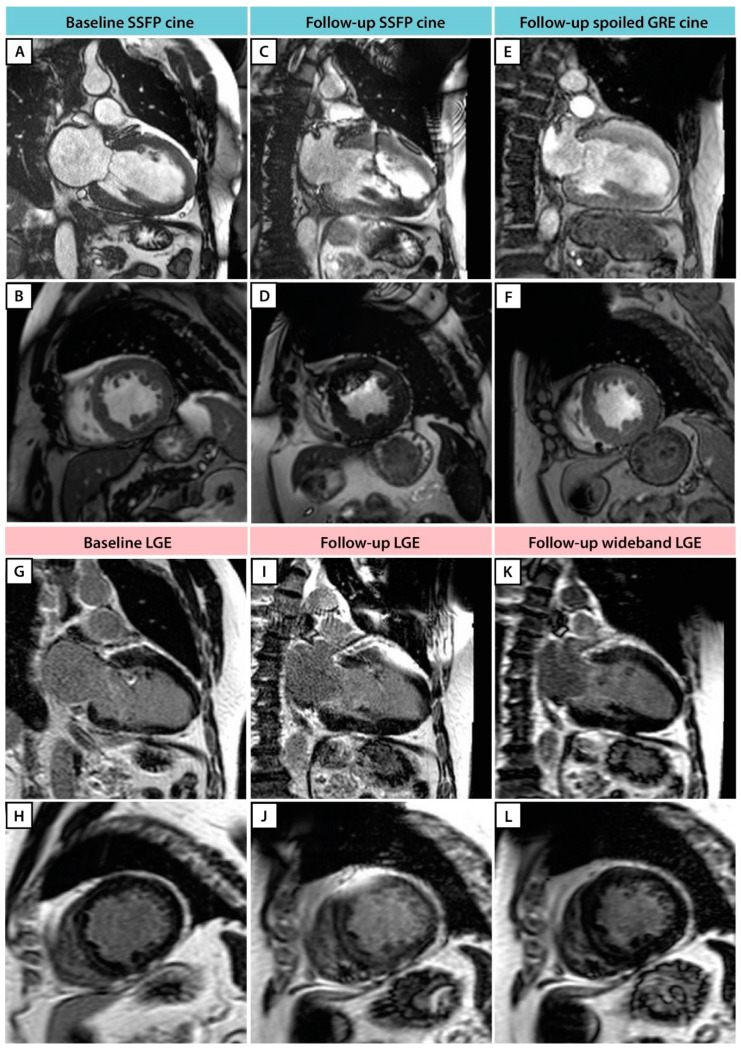
Patient example: CMR cine and LGE images at baseline and follow-up. Image quality display of the two-chamber and mid-short axis cine imaging at baseline (**A**,**B**), follow-up using the SSFP technique (**C**,**D**), and follow-up using the spoiled gradient echo (GRE) technique (**E**,**F**). Image quality display of two-chamber and mid-short axis LGE imaging at baseline (**G**,**H**), follow-up using the conventional LGE (**I**,**J**), and follow-up using the wideband LGE technique (**K**,**L**).

In the present study, some CRT generator-related signal loss remained in the LV anterior wall segments on the cine images, even when using a spoiled gradient echo-based sequence (Figure 5). However, it is worth highlighting that there was no significantly disruptive image artefact observed in the lateral wall, which would correspond to a lead-induced artefact originating from the LV coronary sinus lead [18]. On post-implant LGE images, extensive hyper-intensity artefacts were often present in the LV anterior region using the regular LGE sequence. However, the implementation of novel wideband LGE techniques can greatly eliminate these artefacts and significantly improve image quality, especially in patients with a CRT-D. Although wideband LGE was not part of the initial study protocol, the installation of a wideband LGE sequence before the last study patient resulted in less notable hyper-intensity artefacts (as demonstrated in Figure 5) and significantly improved image quality. The application of this wideband LGE sequence would have significant implications for electrophysiology procedures (ventricular tachycardia ablation) in patients with ICDs, as accurate and artefact-free imaging is crucial for precise diagnosis, treatment planning, and guidance during interventions. Altogether, the technical advancements in both CRT settings during CMR and CMR image quality enable the accurate evaluation of resynchronization therapy and follow-up in CRT patients.

### 4.2. Acute Hemodynamic Changes and Reverse Remodeling during Follow-up

In this study, evidence was obtained for reverse LV remodeling as early as six weeks after CRT implantation by observing improvement in LV volumes at follow-up during intrinsic rhythm as compared to the baseline assessments [19]. It has been demonstrated that reverse LV remodeling after CRT is associated with a favorable long-term outcome [20,21]. Therefore, CMR assessment post-device implantation allows for the determination of a detailed response after implantation and subsequently contributes to estimating the clinical prognosis [22].

Moreover, an acute deterioration in LV pump function mediated by dyssynchrony and discoordination was observed by turning off BIV pacing during the follow-up scan. This finding underlines that BIV pacing during CMR is crucial for the proper assessment of LV hemodynamics and can be useful in the identification of non-responders who might benefit from CRT optimization.

### 4.3. Device Optimization

Optimizing cardiac function can be achieved by individualized programming of the pacing configuration and timing delays [23,24]. Accordingly, the maximization of hemodynamic benefits can be accomplished by invasive hemodynamic testing using a conductance catheter [9]. Acquiring simultaneous pressure and volume measurements throughout the cardiac cycle can provide a comprehensive assessment of the LV pump function. Unfortunately, the accessibility of PV loop measurements is limited, and invasive hemodynamic optimization is largely unfeasible in clinical practice. A non-invasive and reproducible alternative strategy for device optimization is currently lacking [25]. Additionally, we did not find a significant relationship between the reduction in QRS duration and hemodynamic response at follow-up. This suggests that while QRS duration is often used as a marker for response to CRT, it may not always correlate with improved hemodynamics.

In our study, we sought to explore the potential of CMR as a non-invasive alternative for device optimization. CMR-based optimization can overcome the inherent limitations of invasive hemodynamic testing and may serve as a promising future alternative [26]. Moreover, CMR is widely regarded as providing more accurate cardiac evaluation compared to echocardiography, particularly when aiming for a more detailed assessment of myocardial motion abnormalities, such as apical rocking and septal flash, using strain analysis in patients with LBBB.

To assess the agreement between invasively obtained and CMR-derived LV volumes, we compared both strategies during an identical BIV pacing configuration and intrinsic rhythm. Despite the fact that both techniques measure LV volumes in a significantly different manner, our results demonstrate a modest relationship between the two modalities. The dissimilarity can possibly be explained by the different assessment times during the day and especially by differences in heart rate between the CMR study and the invasive procedure. Moreover, the insuperable limitations of the conductance catheter can also contribute to the differences measured using both techniques, as elucidated by Vernooy et al. [27]. Positioning of the catheter in the middle of the LV cavity can be challenging, especially in dilated and dyssynchronous hearts, and consequently, the reliability of the volume signal acquisition may be hampered. In this study, we reconstructed a PV loop using an invasively obtained LV pressure curve and an LV volume curve derived from CMR as a proof of concept. However, additional brachial pressure measurements during CMR can conceivably be used to reconstruct completely non-invasive PV loops, as demonstrated by Seemann et al. [28].

Additionally, only two device settings were tested in patients implanted with a CRT-D, and the study focus was predominantly on feasibility. The CRT-D device used in the present study is currently the only one allowing BIV pacing in the MRI-safe mode. Unfortunately, this MRI safe mode comprises a restricted BIV pacing option, with non-variable AV delays and pacing configurations. This could hamper the evaluation of the response on CRT by possibly underestimating the patients’ cardiac function. Nevertheless, we have shown a response in LV function and mechanics upon turning off BIV pacing. More research is needed to test whether more subtle changes in BIV pacing settings, such as pacing configurations and AV delay variation, demonstrate a clear response in CMR-derived hemodynamic parameters. Furthermore, research involving other device manufacturers to determine the generalizability of the results would enhance the overall validity and applicability of the study’s results.

## 5. Limitations

This pilot study is an initial step in exploring the feasibility of CMR imaging during BIV pacing. Inherent to its setup, the study has a small sample size. Furthermore, as the MRI safe mode did not include AOO pacing as an option, the CRT-off option was used to mimic the AOO mode. As a result, there was a difference in heartrate (>10 bpm) between the BIV pacing mode and the CRT-off mode, potentially affecting diastolic filling time and cardiac function [29]. Lastly, the intrinsic rhythm during CMR and invasive measurements could differ (due to stress during the invasive procedure or anxiety during CMR), as these are two separate measurements.

## 6. Conclusions

Post-CRT implantation CMR assessing acute pump function changes in regards to different CRT-pacing settings is feasible and provides important insights into the effects of BIV pacing on cardiac function and contraction patterns. Post CRT-implantation assessment using CMR may constitute a future CRT optimization strategy.

## Figures and Tables

**Table 1:** Baseline characteristics of the study population.

Parameter	*n* = 10
Demographics	
Age in years	70 ± 7
Sex, male/female	9/1
BMI	28.3 ± 6.9
BSA	2.1 ± 0.3
Hypertension (%)	4 (40%)
NYHA II/III	1/9
Etiology, ICMP/NICMP	4/6
Ischemic LV LGE pattern (%)	3 (30%)
Non-ischemic LV LGE pattern (%)	3 (30%)
QRS duration (ms) LBBB	167 ± 15 10 (100%)
CRT-D (%)	10 (100%)
Left sided pre-pectoral pocket (%)	10 (100%)
Medications	
ACE inhibitors, ARBs or ARNi (%)	10 (100%)
Beta-blockers (%)	10 (100%)
Diuretics (%)	9 (90%)

Values are expressed as number (percentage), mean ± SD, or median [25th–75th percentile]. ACE, angiotensin-converting enzyme; ARB, angiotensin receptor blocker; ARNi, angiotensin receptor-neprilysin inhibitor; BMI, body mass index; BSA, body surface area; CRT-D, cardiac resynchronization therapy defibrillator; ECG, electrocardiogram; ICMP, ischemic cardiomyopathy; LBBB, left bundle branch block; LGE, late gadolinium enhancement; LV, left ventricle; NICMP, non-ischemic cardiomyopathy; NYHA, New York Heart Association.

**Table 2:** Patient CMR results.

	Baseline	FU–BIV Pacing	FU-Intrinsic Rhythm	Baseline vs. FU–BIV Pacing, *p*-Value	Baseline vs. FU–Intrinsic Rhythm, *p*-Value	FU–BIV Pacing vs. FU–Intrinsic Rhythm, *p*-Value
Heart rate (bpm) *LV volumetric function*	68 ± 9	81 ± 8	65 ± 11	**<0.01**	0.33	**<0.001**
ESV (mL)	253.8 ± 41.3	161.4 ± 36.3	194.9 ± 37.1	**<0.001**	**<0.001**	**<0.01**
EDV (mL)	336.9 ± 52.8	236.2 ± 31.8	268.1 ± 42.3	**<0.001**	**<0.001**	**<0.01**
LVEF (%)	24.6 ± 5.6	32.2 ± 8.7	27.4 ± 5.9	**0.04**	0.28	**<0.01**
*LV strain and dyssynchrony*						
GLS (%)	−8.2 ± 2.1	−6.2 ± 1.6	−7.9 ± 2.2	0.06	0.74	**0.02**
GCS (%)	−6.2 ± 3.9	−7.8 ± 3.0	−7.2 ± 2.4	0.35	0.53	0.18
GRS (%)	8.2 ± 4.6	10.3 ± 4.9	8.9 ± 3.5	0.37	0.74	0.08
Peak_septal_ - peak_lateral_ delay (ms)	258 ± 121	57 ± 46	183 ± 86	**<0.001**	**0.04**	**<0.01**
Septal systolic rebound stretch (%)	6.1 ± 3.1	2.1 ± 2.6	6.0 ± 3.0	**0.01**	0.93	**<0.01**

Values are expressed as mean ± SD. BIV, biventricular; Bpm, beats per minute; CMR, cardiovascular magnetic resonance imaging; EDV, end diastolic volume; EF, ejection fraction; ESV, end systolic volume; FU, follow-up; GLS, global LV longitudinal strain; GCS, global circumferential strain; GRS, global radial strain; LV, left ventricular; LVEF, left ventricular ejection fraction.

## Data Availability

The data underlying this article will be shared upon reasonable request to the corresponding author.

## Supplementary Materials

The following supporting information can be downloaded at: https://www.mdpi.com/article/10.3390/jcm12123998/s1, Figure S1: PICARIA-CRT CMR scan protocol; Figure S2: CMR image quality score form; Figure S3: Post-device implantation mid-short axis cine image of all patients; Table S1: Post-device implantation CMR scan quality.

## Abbreviations

AOO	atrial asynchronous mode
BIV pacing	biventricular pacing
CMR	cardiac magnetic resonance
CRT	cardiac resynchronization
EDV	end-diastolic volume
EF	ejection fraction
ESV	end-systolic volume
FU	follow-up
LGE	late gadolinium enhancement
LV	left ventricle
SRS_septal_	systolic rebound stretch of the septum
