# Supplementary material for: Feasibility of CMR Imaging during Biventricular Pacing: Comparison with Invasive Measurement as a Pathway towards a Novel Optimization Strategy
Source: J Clin Med. 2023 Jun 12;12(12):3998. doi: 10.3390/jcm12123998 (PMC10298880; doi:10.3390/jcm12123998)
Supplement: Supplementary file 1 [file jcm-12-03998-s001.zip › jcm-2426361-supplementary.pdf]

Feasibility of CMR Imaging during Biventricular Pacing: *Comparison with Invasive Measurements as a Pathway towards a Novel Optimization Strategy*

Luuk H.G.A. Hopman[1], Msc; Alwin Zweerink[1], MD, PhD; Anne-Lotte C.J. van der Lingen[1], MD; Marthe J. Huntelaar[1], Msc; Mark J. Mulder[1], MD; Lourens F. H. J. Robbers[1], MD, PhD; Albert C. van Rossum[1], MD, PhD; Vokko P. van Halm[1], MD, PhD; Marco J.W. Götte[1], MD, PhD; Cornelis P. Allaart[1], MD, PhD

[1]Department of Cardiology, Amsterdam UMC, Amsterdam, The Netherlands

Supplemental tables:

Table S1. Post-device implantation CMR scan quality

Supplemental figures:

Figure S1: PICARIA-CRT CMR scan protocol

Figure S2: CMR image quality score form

Figure S3: Post-device implantation mid-short axis cine image of all patients

Table S1. Post-device implantation CMR scan quality.

**CMR cine image quality after CRT-D implantation**																
**Segment**	**1**	**2**	**3**	**4**	**5**	**6**	**7**	**8**	**9**	**10**	**11**	**12**	**13**	**14**	**15**	**16**
Reader 1																
Mean value	2.2	1.4	1	1	1.1	1.7	2	1.2	1	1	1	1.4	1.1	1	1	1
#1 (no artefacts)	4	8	10	10	9	7	5	9	10	10	10	8	9	10	10	10
#2 (limited artefacts)	3	0	0	0	1	0	1	0	0	0	0	0	1	0	0	0
#3 (substantial artefacts)	0	2	0	0	0	2	3	1	0	0	0	2	0	0	0	0
#4 (extensive artefacts)	3	0	0	0	0	1	1	0	0	0	0	0	0	0	0	0
Reader 2																
Mean value	2.1	1.1	1.1	1	1	1.7	1.7	1.1	1	1	1	1.1	1.2	1.2	1	1
#1 (no artefacts)	5	9	9	10	10	6	5	9	10	10	10	9	8	8	10	10
#2 (limited artefacts)	2	1	1	0	0	2	3	1	0	0	0	1	2	2	0	0
#3 (substantial artefacts)	0	0	0	0	0	1	2	0	0	0	0	0	0	0	0	0
#4 (extensive artefacts)	3	0	0	0	0	1	0	0	0	0	0	0	0	0	0	0
Overall cine image score of both readers	2.2	1.3	1.1	1	1.1	1.7	1.9	1.2	1	1	1	1.3	1.2	1.1	1	1

**CMR LGE image quality after CRT-D implantation**																
**Segment**	**1**	**2**	**3**	**4**	**5**	**6**	**7**	**8**	**9**	**10**	**11**	**12**	**13**	**14**	**15**	**16**
Reader 1																
Mean value	3	2.5	1	1	1.2	2.3	3.6	2.9	1	1	1.2	2.9	2.4	1.7	1	1.6
#1 (no artefacts)	2	3	10	10	9	4	1	2	10	10	9	2	4	7	10	7
#2 (limited artefacts)	1	1	0	0	0	1	0	0	0	0	0	0	1	0	0	0
#3 (substantial artefacts)	2	4	0	0	1	3	1	5	0	0	1	5	2	2	0	3
#4 (extensive artefacts)	5	2	0	0	0	2	8	3	0	0	0	3	3	1	0	0
Reader 2																
Mean value	2.9	1.9	1.2	1	1.1	2	3.5	2.4	1.2	1.1	1.1	2.5	3.3	2	1	1.6
#1 (no artefacts)	2	5	9	10	9	5	1	2	9	9	9	2	1	3	10	5
#2 (limited artefacts)	2	2	0	0	1	1	0	4	0	1	1	3	1	5	0	4
#3 (substantial artefacts)	1	2	1	0	0	3	2	2	1	0	0	3	2	1	0	1

#4 (extensive artefacts)	5	1	0	0	0	1	7	2	0	0	0	2	6	1	0	0
Overall LGE image score of both readers	3.0	2.2	1.1	1	1.2	2.2	3.6	2.7	1.1	1.1	1.2	2.7	2.9	1.9	1	1.6

Figure S1: PICARIA-CRT CMR scan protocol.

MRI Scan Protocol PICARIA-CRT

Pre-implantation MRI protocol

**1.**	**LV Function**
	SSFP Cine long axis 3 standard views (4ch, 3ch, 2ch).
ADMINISTER 0.15MMOL/KG CONTRAST AGENT	
**2.**	**LV Function**
	SSFP Cine short axis stack LV coverage; 5mm/100% gap, temp resolution 20ms.
**3.**	**Late Gadolinium Enhancement Segmented**
	10-15 min after contrast administration, long/short axis LV coverage; copy cine orientation.

Post-implantation MRI protocol

**1.**	**LV Function**
	Cine long axis 3 standard views (4ch, 3ch, 2ch) and short axis LV stack coverage; 5mm/100% gap, temp resolution 20ms
TURN OFF CRT	
**2.**	**LV Function**
	Cine long axis 3 standard views (4ch, 3ch, 2ch).
ADMINISTER 0.15MMOL/KG CONTRAST AGENT	
**3.**	**LV Function**
	Cine short axis LV slack coverage; 5mm/100% gap, temp resolution 20ms.
**4.**	**Late Gadolinium Enhancement Segmented**
	10-15 min after contrast administration, long/short axis LV coverage; copy cine orientation.

PICARIA-CRT

Figure S2: CMR image quality score form.

CMR image quality in CRT-D patients

1 = No image artefact at all and no limitation in image interpretation.

2 = Good image quality with limited artefacts (affecting less than 25% of the ventricles or the defined segment).

3 = Poor image quality with substantial artefacts (affecting more than 25% but less than 75% of the ventricles or the defined segment).

4 = Meaningful image assessment impossible due to extensive artefacts (affecting more than 75% of the ventricles or the defined segment).

Segment	SHORT AXIS CINE:	LGE:
1	① ② ③ ④	① ② ③ ④
2	① ② ③ ④	① ② ③ ④
3	① ② ③ ④	① ② ③ ④
4	① ② ③ ④	① ② ③ ④
5	① ② ③ ④	① ② ③ ④
6	① ② ③ ④	① ② ③ ④
7	① ② ③ ④	① ② ③ ④
8	① ② ③ ④	① ② ③ ④
9	① ② ③ ④	① ② ③ ④
10	① ② ③ ④	① ② ③ ④
11	① ② ③ ④	① ② ③ ④
12	① ② ③ ④	① ② ③ ④
13	① ② ③ ④	① ② ③ ④
14	① ② ③ ④	① ② ③ ④
15	① ② ③ ④	① ② ③ ④
16	① ② ③ ④	① ② ③ ④

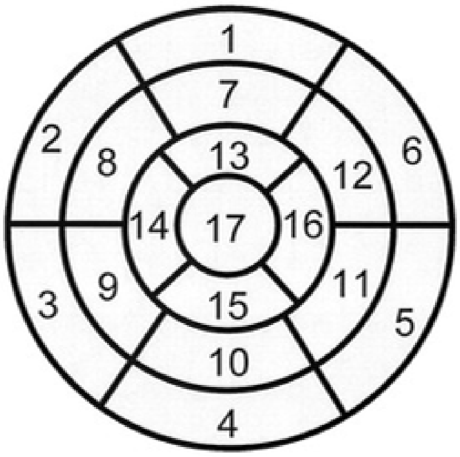

Evaluated by: ____________

Study ID: _________

Remark:

Figure S3: Post-device implantation mid-short axis cine image of all patients.

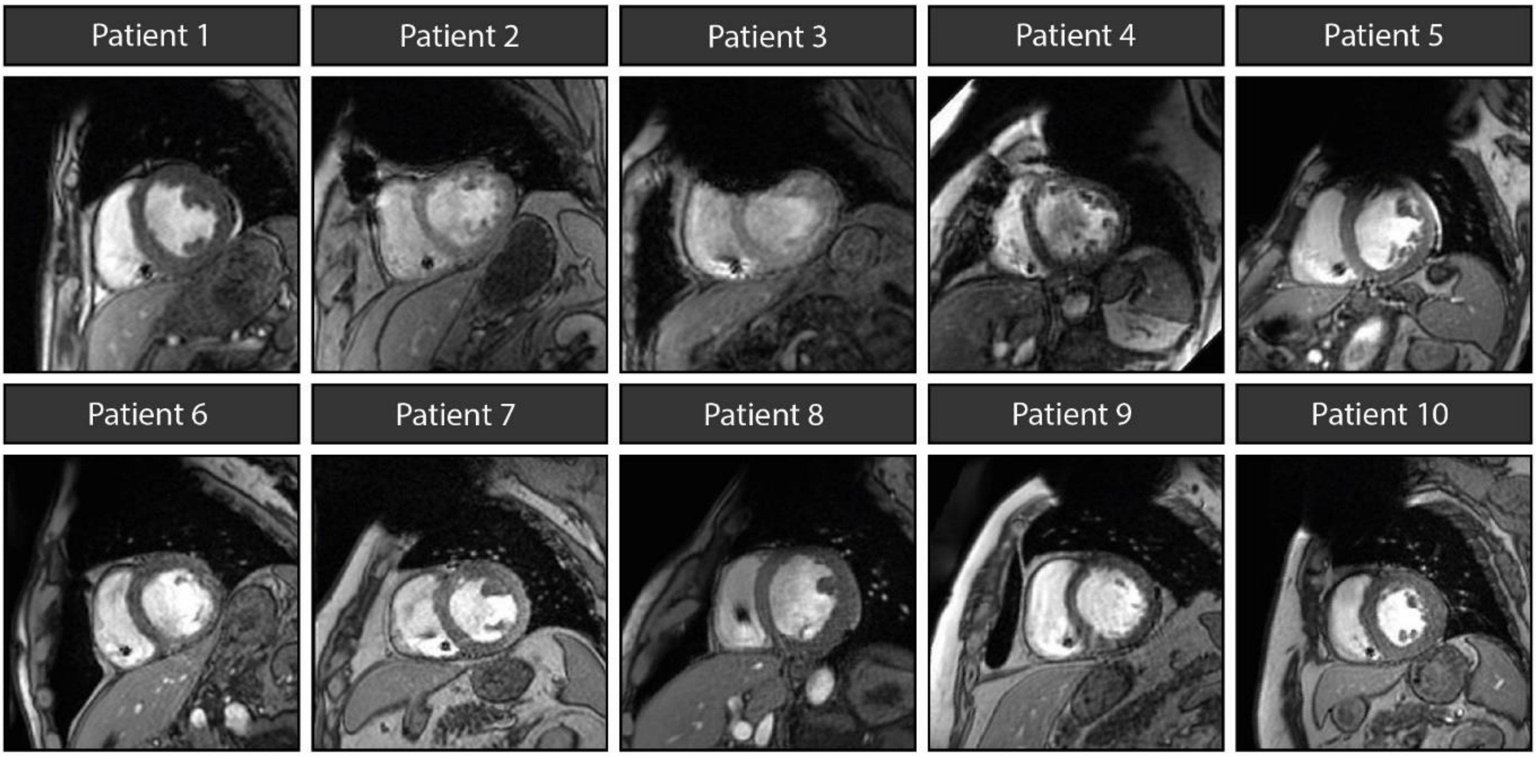

Mid-slice spoiled gradient echo cine images (post-CRT implantation) in end-diastole of all 10 study patients. Device artefact is sometimes present in the anterior wall region of the left ventricle (most prominent in patient 3 and 4).
